# Supplementary material for: Systematical assessment of the impact of single spike mutations of SARS-CoV-2 Omicron sub-variants on the neutralization capacity of post-vaccination sera
Source: Front Immunol. 2023 Nov 10;14:1288794. doi: 10.3389/fimmu.2023.1288794 (PMC10667444; doi:10.3389/fimmu.2023.1288794)
Supplement: Supplementary file 1 [file DataSheet_1.pdf]

Supplementary information

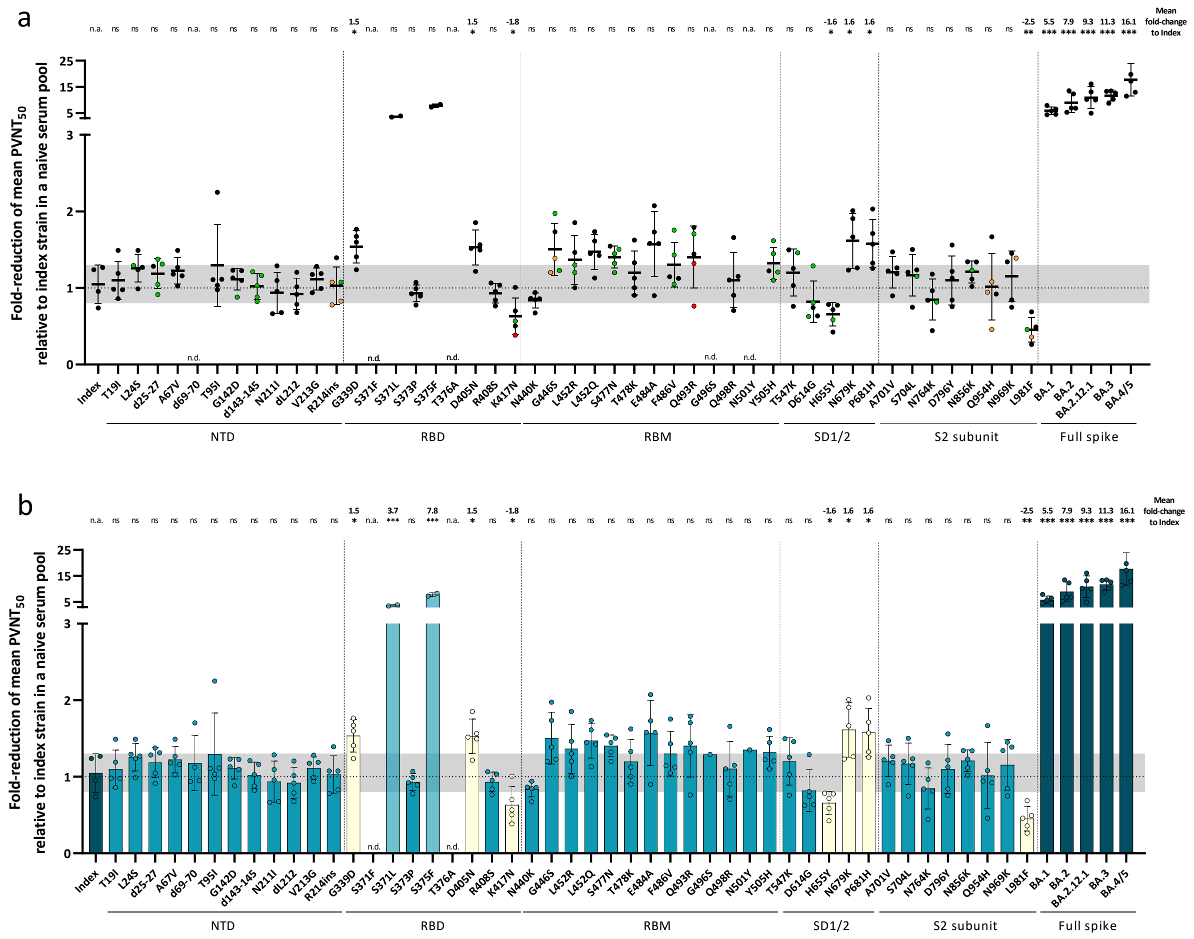

Supplementary figure 1

a. Fold-reduction of the mean $PVNT_{50}$s from VOC and single mutant pseudo-viruses relative to the index strain were measured in a pool of six serum samples from boosted infection-naive individuals. No data was obtained for mutants with too low virus titers because of technical limitations (indicated as n.d.). Single $PVNT_{50}$s are color coded according to the quantity of virus input used in each experiment. Virus input > 300 ffu/well (black), 200 – 300 ffu/well (green), 100 – 200 ffu/well (orange), < 100 ffu/well (red). Respective spike domains of mutants are indicated below the x-axis. **b**. Fold-reduction of the mean $PVNT_{50}$s from VOC (dark petrol) and single mutant (petrol) pseudo-viruses relative to the index strain were measured in a pool of six serum samples from boosted infection-naive individuals. Raw neutralization data are provided in the supplements. Mutants with potentially biased significant fold-reductions are shown in light petrol. No data was obtained for mutants with too low virus titers because of technical limitations (indicated as n.d.). Respective spike domains of mutants are indicated below the x-axis. Bars and whiskers present the mean fold-reduction ± standard deviation. Standard deviation of the index fold-reductions are shown as a light grey horizontal bar. Single dots represent single measured fold-reductions. Significant fold-reductions, as well as significance levels are provided on the top of the graph. Statistical analysis was performed using the Brown-Forsythe and Welch ANOVA test without correction for multiple comparisons. Statistical significance was defined by a value of * < 0.05; ** < 0.01; *** <0.001. n.s. is not significant; n.a. is not available.

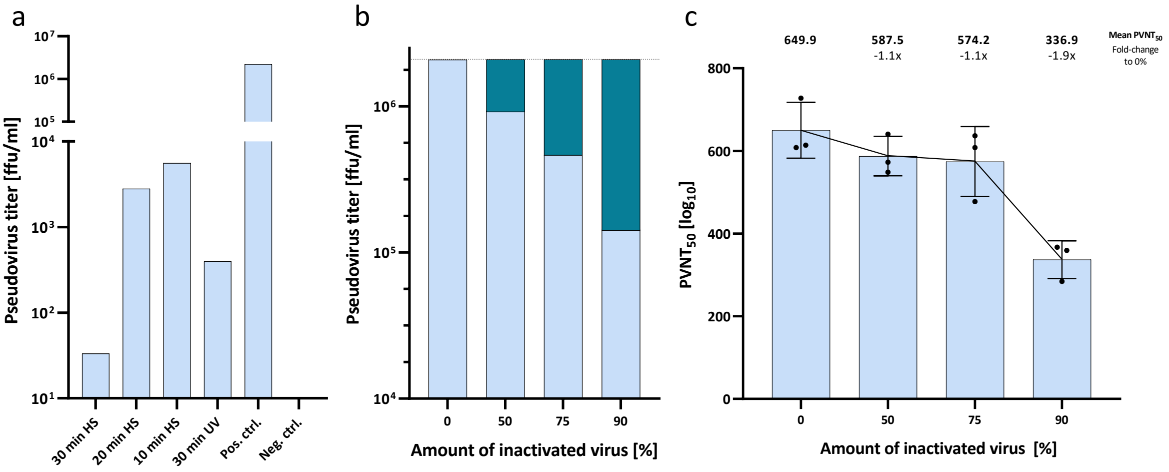

<u>Supplementary figure 2</u>

Pseudo-virus titers measured after different inactivation protocols and using varying amounts of inactivated virus. **a**. shows the pseudo-virus titers in ffu/ml after heat (HS)- or UV-inactivation. Bars present the total pseudo-virus titer determined by the number of infected VeroE6 cells. **b**. shows the pseudo-virus titer of pseudo-virus stocks containing 0%, 50%, 75%, or 90% of UV-inactivated index pseudo-virus (light blue). The pseudo-virus titer that was supposedly reduced by UV-inactivation is shown as petrol columns for each virus stock up to the titer of the index virus stock with 0% UV-inactivated virus (indicated as dashed horizontal line). **c**. $PVNT_{50}$s against index pseudo-virus containing equal infective viral particles with 0%, 50%, 75%, or 90% of UV-inactivated index pseudo-virus. Mean $PVNT_{50}$s are plotted and shown on top of the graph. Fold-change relative to the virus containing no UV-inactivated index virus is shown below the $PVNT_{50}$s. Bars and whiskers present the mean $PVNT_{50}$ ± standard deviation. Single dots indicate average neutralization titers from three independent technical replicates.

Supplementary table 1 Study characteristics of all studies from the systematic literature review.

Study First Author	Journal	Date	Analyzed mutations	Specimen
Pastorio[1]	Cell Host & Microbe	14.09.2022	G339D, S373P, S375F, K417N, N440K, S477N, T478K, E484A, Q493R, Q498R, N501Y, Y505H, H655Y, N679K, P681H, N764K, D796Y, Q954H, N969K, A67V, d69-70, T95I, d142-144, R214EPE, S371L, G446S, G496S, T547K, N856K, L981F, T19I, G142D, V213G, S371F, T376A, D405N, R408S	Serum
Wang[2]	Nature	10.02.2021	K417N, N501Y	Plasma
Cao[3]	Nature	21.05.2021	K417N, N501Y	Plasma/ serum
Wang[4]	CellPress Immunity	13.07.2021	K417N, N501Y	Plasma
Neerukonda[5]	Viruses	11.12.2021	T478K, K417N, L452R	Serum
Alenquer[6]	PLOS Pathogens	05.08.2021	N501Y, S477N, L452R,	Serum
Ferreira[7]	Journal of Infectious Diseases	09.09.2021	L452R	Serum
Zhang[8]	Emerging Microbes & Infections	07.04.2022	L452R, T478K, G142D	Serum
Wang[9]	Emerging Microbes & Infections	21.12.2021	L452Q	Serum
Wu[10]	Frontiers	17.06.2021	S477N	Serum
Kuzmina[11]	Viruses	13.04.2022	P681H, A701V, N501Y, L452R, S477N	Serum
Tada[12]	American Society for Microbiology	01.06.2021	d69-70, N501Y	Plasma
Xie[13]	Nature medicine	08.02.2021	N501Y	Serum

Supplementary table 2 Detailed participant characteristics. N/A not applicable; BNT162b2 (BNT), AZD1222 (VAX), mRNA-1273 (MOD)

	Sex	Age group	Baseline SARS-CoV-2 status	Vaccine type	Interval: dose 1-2	Interval: dose 2-3	Interval: dose 3-4	Interval: last dose until serum collection	Interval: last dose until infection	Interval: infection until serum collection	Severity (WHO grade)	Prevalent variant	Date of infection
1st Boost, naive	m	30-40	negative	BNT+BNT+BNT	3 weeks	42 weeks	N/A	29 weeks	N/A	N/A	N/A	N/A	N/A
	f	30-40	negative	VAX+BNT+BNT	10 weeks	28.5 weeks	N/A	32 weeks	N/A	N/A	N/A	N/A	N/A
	f	50-60	negative	VAX+VAX+BNT	10 weeks	26 weeks	N/A	32 weeks	N/A	N/A	N/A	N/A	N/A
	f	20-30	negative	BNT+BNT+BNT	6 weeks	32.5 weeks	N/A	29 weeks	N/A	N/A	N/A	N/A	N/A
	m	50-60	negative	BNT+BNT+MOD	6 weeks	22 weeks	N/A	35 weeks	N/A	N/A	N/A	N/A	N/A
	f	20-30	negative	BNT+BNT+BNT	4 weeks	45.7 weeks	N/A	39 weeks	N/A	N/A	N/A	N/A	N/A
	f	50-60	negative	BNT+BNT+BNT	6 weeks	25.5 weeks	N/A	31.5 weeks	N/A	N/A	N/A	N/A	N/A
	m	20-30	negative	BNT+BNT+BNT	6 weeks	28 weeks	N/A	31.5 weeks	N/A	N/A	N/A	N/A	N/A
	m	50-60	negative	BNT+BNT+BNT	6.5 weeks	26 weeks	N/A	36.5 weeks	N/A	N/A	N/A	N/A	N/A
1st Boost, breakthrough	f	30-40	positive, no PCR	BNT+BNT+MOD	3 weeks	21 weeks	N/A	28 weeks	13 weeks	16 weeks	Mild	BA.2	March 2022
	f	20-30	positive (confirmed)	BNT+BNT+BNT	6 weeks	21 weeks	N/A	29.5 weeks	1.5 weeks	28 weeks	Mild	BA.1/ BA.2	January 2022
	f	20-30	positive, no PCR	BNT+BNT+BNT	6 weeks	21 weeks	N/A	30 weeks	26 weeks	3.5 weeks	Mild	BA.5	June 2022
	f	20-30	positive (confirmed)	VAX+BNT+BNT	12 weeks	24 weeks	N/A	29 weeks	6 weeks	24 weeks	Mild	BA.1/ BA.2	January 2022
	m	30-40	positive, no PCR	BNT+BNT+BNT	6 weeks	28 weeks	N/A	26.5 weeks	23.5 weeks	3 weeks	Mild	BA.5	July 2022
	m	30-40	positive (confirmed)	VAX+BNT+BNT	11.5 weeks	29 weeks	N/A	28 weeks	24 weeks	3 weeks	Mild	BA.5	July 2022
	f	50-60	positive (confirmed)	BNT+BNT+MOD	6 weeks	20 weeks	N/A	30.5 weeks	14 weeks	16.5 weeks	Mild	BA.2	March 2022
	m	50-60	positive (confirmed)	VAX+BNT+MOD	6 weeks	20 weeks	N/A	33 weeks	28 weeks	3 weeks	Mild	BA.5	July 2022
	m	20-30	positive, no PCR	BNT+BNT+BNT	4 weeks	24 weeks	N/A	37 weeks	21 weeks	12 weeks	Mild	BA.2	March 2022
	f	30-40	positive, no PCR	BNT+BNT+BNT	5 weeks	23 weeks	N/A	40 weeks	16 weeks	18 weeks	Mild	BA.2	March 2022
2nd Boost, naive	m	20-30	negative	MOD+MOD+BNT+BNT	6 weeks	24 weeks	10 weeks	23 weeks	N/A	N/A	N/A	N/A	N/A
	f	20-30	negative	BNT+BNT+BNT+BNT	7 weeks	25 weeks	24 weeks	4 weeks	N/A	N/A	N/A	N/A	N/A
	f	50-60	negative	BNT+BNT+BNT+BNT	4 weeks	27 weeks	32.5 weeks	3 weeks	N/A	N/A	N/A	N/A	N/A
	f	20-30	negative	BNT+BNT+BNT+BNT	4 weeks	45.7 weeks	39.5 weeks	1.5 weeks	N/A	N/A	N/A	N/A	N/A
